# Operative Surgeons.—The Great Untaught

**Published:** 1894-08-25

**Authors:** 


### The Hospital

An Institutional Journal of

The Medical Sciences and Hospital Administration, with

Vol. XVI.—No. 413.] AN EXTRA NURSING SUPPLEMENT. [Aug. 25, 1894.

### Operative Surgeons—The Great Untaught.

"The operative art of surgery is truly a high art; the demands it makes on the hand, the eye, and the brain of the artist are the same for surgery and the fine arts. Surgery and fine art may well be practised by the same man. . . . The art in etching of Sir Seymour Haden, and the art in surgery of Sir Henry Thompson, each supreme in its way, have two sides on the surface only: at bottom they are identical. . . . The world lost a great surgeon in Leonardo da Vinci, but it gained a great painter. In Sir Charles Bell it lost a great artist and gained a great surgeon. Now of this art of surgery—Where and what is the teaching? Where and who are the masters who guide and correct the young artists in their work? It may be said that of masters there are plenty, but they do not teach. There is no real teaching of the art of surgery in this country."

This passage, quoted from the address on surgery of Mr. Greig-Smith to the members of the British Medical Association at their recent Bristol Congress, is a text which requires no sermon for its elucidation. What it does require is an honest *mea culpa, mea gravissima culpa*, from every honest professor of surgery throughout this land. To say that operative surgery is an art of supreme importance which can be best learnt when it is adequately taught is a proposition of the most obvious kind. To say, on the other hand, that it is not taught at all in a country like our own, which prides itself most of all upon its science, its practice, and its modernity, is a statement which will, at least, astonish the laity whatever may be thought of it by those members of the medical profession, in whom familiarity with medicine may have bred contempt. Happily for the honour of our great profession, when Mr. Greig-Smith declared that the art of operative surgery was not taught, he did not affirm at the same time that it was not skilfully practised. As a matter of fact, it is skilfully practised, and that because British operating surgeons are, as a class, men of character, intellect, and culture; and finding out, as they soon do by practice, the absolute necessity for a high degree of operative skill, they set themselves, each in his own way, to acquire that skill as speedily and competently as possible. So that our surgeons, however their technical training may have been neglected, have, as a class, attained to a very meritorious degree of operative efficiency.

But the contention is that modern surgery is, absolutely and in itself, a great art, whilst in its bearing upon the happiness and bodily well-being of the whole community, it is an art of supreme importance. The average layman would say that to construct an argument on behalf of the teaching of such an art would be an absurd waste of time. Such an argument cannot by any possibility be needed in a community consisting of persons of average common sense. To which our reply is that we have it on the authority of Mr. Greig-Smith, himself a professor of surgery, and a very learned one, that, as a matter of actual fact, "there is no real teaching of the art of surgery in this country." Though, then, no argument may be needed to show that the art of surgery ought to be taught, it is clear that something is needed to bring about the actual and universal teaching of that art which so obviously ought to be taught, but is not. If operative surgery is not taught, why is it not taught? If it has not hitherto been taught, that lack of teaching stands to our discredit, we had almost said to our disgrace, as a profession; and the disgrace must now be wiped away without further argument or loss of time.

In attempting to remove a discreditable incubus of this sort from the fair fame of our profession one places one's lever upon the fulcrum of manifest and unassailable common sense. But in what direction the lever may be most profitably applied is not quite so obvious. Should our appeal be addressed to the professors of the surgical art, or not rather to those numerous pupils who, one assumes, would fain be taught technique, but cannot come at any systematic teaching? On a subject of this kind the laity themselves have a right to be heard. One can well imagine them standing aghast at the bare idea that the young surgeon who is so manifestly alive and up-to-date in everything that appertains to the science of his profession is but a mere untrained bungler and raw learner in the art and craft of his calling. Little do those members of the laity who pay the young man so liberally suspect that they are being used as raw material for his first crude attempts. The laity, if they did but know, would soon make their demands for the teaching of the surgical art effectual; for they would not employ on their own bodies any surgeon who could not furnish convincing evidence of adequate technical training and competent craftsmanship. There are those who think that the proper persons to bring about this absolutely imperative reform are the senior students and young practitioners who are beginning to realise the scantiness of their practical equipment for the stern competitive battle of medical science and practice which lies before them as the chosen work of their lives.

It is said, apparently with some truth, that the young practitioner is himself chiefly in fault in his own want of technical training. As Mr. Greig-Smith says, "We cannot teach unqualified men the technique of operative surgery, because the unqualified are not permitted to operate. We must begin our teaching after the student is qualified." And here, *mirabile dictu*, the whole difficulty appears to begin. The qualified man, because he is "qualified," will not condescend to be taught. "He was our pupil yesterday," says Mr. Greig-Smith, "but to-day he is our colleague and equal." We must not teach him, even for his own good. This view of the matter ought to be incredible. If it be in any sense true, then medical education produces in some instances this extraordinary result, that it leaves a young man more foolish and more mentally undisciplined than it found him as a raw schoolboy. As a schoolboy he was at least aware of his own ignorance and incompetence. Now he does not even appear to know that for practical purposes he is a mere beginner, and needs nothing so much as the experienced guidance of a practical person to show him how to become laboriously practical too. If this be the condition of mind of any considerable number of our newly qualified practitioners, there must have been something very far from competent in the teaching of their teachers. The finest of all the results of education is that which makes a man realise what he does not know, and what there is yet to be learnt. If this result be not obtained in a study like that of medicine, which is so wide and peep that no mortal man can ever by any possibility master it in all its breadths and depths, there must, we insist, be something fundamentally deficient about the intellectual capacity of our medical teachers.

Perhaps then, after all, it is the teachers of surgery who must be held responsible for the painful and deeply discreditable fact that "there is no real teaching of the art of surgery in this country." They may make this excuse for themselves, that since they were not specially taught and yet by practice have become competent, there is no reason why future generations of untaught men should not become competent likewise. If that is their excuse it is a poor and a mean one. Do they comprehend the greatness and the difficulty of their own art? Do they realise its practical importance to the world? If they do, then surely this realisation imposes upon them the obligation, the sacred obligation, of taking whatever measures may be necessary for the perfecting of their art, and for the conservation and secure transmission of it to future generations. Can the art of surgery, can any real art, be transmitted by means of books or pictures? Can it be transmitted in any single way but one? An art like the surgical can only be handed on from person to person, from master to pupil, from one generation of living hands to another. Among the most truly great of all the builders of the fabric of our civilisation, and the most worthy of honour, are those who improve for us the world's practical arts, and who pass them on to succeeding generations more rational and more perfect than they find them. On the other hand, those who find arts in the world into which they are born, and who use them for their own profit and advantage, but who take no steps for their transmission to future ages of mankind are selfish and vulgar, and belong to the common herd. They have not minds that see intelligently nor hearts that purpose generously. What nobler gift can our generation transmit to posterity than a scientific and practically competent surgery? What more dishonourable word could be said of any generation than this, that it was the master of a perfect art in surgery, but that it took no pains to assure the transmission of that art to generations yet unborn?

